# Our Food and Drink

**Published:** 1854-10

**Authors:** 


// HALL'S JOURNAL OF HEALTH.

VOL. I.] OCTOBER, 1854. [NO. X.

### OUR FOOD AND DRINK.

It is worth the effort of a lifetime, to be able to die well; to die without pain, and in a well-grounded hope of happiness beyond. To die without pain, we must live in health, and live a long time. As a very general rule, those who live to great age, pass away without apparently suffering, as if they were going to sleep. The great secret of a long and healthful life, lies in the judicious use of what we eat and drink. What is "*judicious*," we propose to discuss; but not in such a way as to dictate dogmatically what this or that one shall use, but to let each one decide for himself, under the guidance of a few general principles, founded on observed facts, not on imagined fallacies.

On the sixth day of June, eighteen hundred and twenty-two, a robust, hearty French Canadian of eighteen years, was accidentally shot in the left side; the wound healed, but left an opening in the stomach, which allowed the physician to see at any time what was passing inside, and for the space of fifteen years, a great variety of experiments were made and observations taken; and, in the light of these we make our way.

In clear, cool, dry weather, a thermometer introduced into the stomach, settled at one hundred degrees *Fahrenheit.* In raw, damp, cloudy weather, it remained stationary at ninety-four.

One point gained, then, is, that the temperature of an empty and healthy stomach in good weather, is about one hundred degrees.

Soon after a meal is eaten, the temperature of the stomach is slightly increased, digestion goes on healthily and well, and in four or five hours, the stomach is empty again. By digestion here we mean that what was eaten, whether meat, bread, vegetables or other food, is gradually changed, until it becomes whitish, and thinnish, and sweetish, like milk; it matters not what we eat, or of how many different kinds, it is the same in color, taste and consistence, that is, when digestion is healthy. When digestion is not perfect, the food ferments, becomes sour, rises in the mouth, generates wind, causes belching, and the like familiar symptoms. Digestion being a process of nature, whatever arrests digestion, is a direct interference with nature, always does wrong, and if persevered in, destroys health and life, inevitably.

It was further observed, that cold water, swallowed during the progress of digestion, instantly arrested it, and the process was not resumed, until the water had been there long enough to be warmed from the temperature at which it was drank, to that of the stomach; or from some forty degrees, to a hundred; to accomplish this, the heat must be abstracted from the general system, chilling it. Strong, robust persons may not feel this, but if a man in feeble health drink cold water at a meal, at all largely, he rises from the table chilly, and soon has fever, while the stomach being kept that much longer at work in digesting the food, loses its vigor, the digestion is imperfect, and the food becomes impure, thus laying the foundation of disease. The inevitable inference from these facts is, that

COLD WATER IS INJURIOUS TO HEALTH, if taken at meals. Injurious to the most robust, if taken largely, and to persons in feeble health, if taken at all, beyond a few swallows at a meal.

I therefore set it down, as a clearly established fact, that *a glass or more of cold water, drank habitually at meals, or soon after, is a pernicious practice, even to the most healthy.*

Injury is done in another manner; water, *or any other* fluid, dilutes the gastric juice, and thus weakens its power to dissolve the food. The amount of gastric juice is not lessened, but its power is diminished by its dilution. The finger will be scalded by dipping it into a vessel of boiling water; but if an equal amount of cold water is added, it may be thrust in with impunity, although there is as much heat in the mass as before; but it is more diffused. A glass of brandy will almost strangle a person not accustomed to it, but if largely diluted, it gives no discomfort, although all the brandy is there that was there before. We have then made another advance, that

*Any kind of fluid largely taken at a meal, or soon after*, is positively injurious to health.

*Largely*, is a relative term. An advance of fifty per cent. in the price of any thing is "large," and when it is remembered that but a few table-spoons of gastric juice are furnished at a meal, a glass of cold water, or two or three cups of coffee or tea, is a "*large*" amount of fluid for one meal. Thus, a standing item of advice to my patients is—*Take but half a glass of water at a single meal, or a single cup of weak coffee or tea, never increasing the strength or quantity, and drink nothing within an hour after eating.*

If cold drinks are injurious at meals, cold food is for the same reason also injurious; thus it is, that some of the most terrible forms of disease are brought on by persistence in eating cold food, exclusively, especially in winter time. If cold fluids are injurious at meal, we naturally conclude that warm fluids, in moderation, are beneficial, and rightly so. The young of the animal creation are furnished with sustenance warmed by nature; and the choice morsel is warmed in the beak of the parent bird, before arriving at the nest of her young. We instinctively, almost, prepare something warm for the weary or the invalid, hence the virtue oft times ascribed to drinking milk, *warm* from the cow, not a very palatable idea, it must be confessed. It then follows, that

*If we drink any thing at meals, it should be first warmed.*

We may safely admit, that the universal custom of a country is founded on common sense, common sense being the teachings of experience. Common consent, and the experience of the civilized world is, that a cup of good hot coffee for breakfast, and a cup of good hot tea for supper, is "*wholesome.*" If a person is prejudiced against "*store tea* and coffee," then any of our common garden herbs may be substituted, as balm, sage, sassafras, and the like; it is the *warmth* that comes first in importance, and there must be the taste of something palatable in it, or the stomach will loathe it. I am well aware that some persons consider tea and coffee poisonous, as did an enthusiastic young "*theological*" at New Brunswick, a few years ago, and demonstrated it, as he thought, to the old Domine, then in his eighty-sixth year, and still an efficient laborer in the vineyard. "It may be a poison as you say," replied the old veteran, as the sly mischief twinkled out of the corner of his eye, "but it must be a very slow poison, for I have taken it regularly night and morning for these eighty years, and am, as you see, not dead yet." The same has been said of Dr. Johnson.

But how comes it that so many sensible people believe that tea and coffee are poisonous? Just as they have come to the adoption of any other fallacy. Somebody who had nothing else to do, imagined it, then hunted up facts and parts of facts to prove it; and what with adding a little to one fact, and suppressing from another, a really plausible case was made out, to every reader or hearer, who had rather admit a statement, than take the trouble thoroughly to sift its truth, and there are many such.

The first temperance lecture I ever heard was before an old-fashioned temperance society, when they allowed us to drink as much cider, and beer, and ale, and wine as we pleased, only the stronger sorts were forbidden, such as whisky, gin, rum, brandy, and the like. The main argument ran as follows: "Spirituous liquors were composed of certain elements, and these elements in larger quantities, were found in the most death-dealing-poisons." We were old enough then to understand, that the air we breathed contained oxygen, add a little more with water, and it became aqua fortis; the shallowness of the argument made such an impression, that we never attended a temperance lecture afterwards, except once to hear Barnum, when it was the man who drew, not the subject. The consequence was, that we have never joined any of the temperance societies of the times, and never will, unless we somerset hugely; not that we are enemies to temperance, or friendly to the habitual use of spirituous liquors as a beverage. But we never have been personally friendly to the "temperance principles," so called. We have always considered it unmanly to give "*the pledge*," anti-Christian,* and inefficient. Unmanly, because a true man, that is, a man in moral courage,

* Not un-Christian, but simply opposed to the spirit of Christianity.

and a man in Christian principle, has always that self-command, which will control his actions at the moment it becomes necessary to act. If it is only the rod of a pledge hanging over him, which restrains him, then is he virtuous not from principle, but from compulsion, and consequently not virtuous at all. My idea of *a true man is, one who is prompted to act right, when the moment for action arrives, purely because it is right, and he loves right acting.*

I have considered it anti-Christian for any church member to join a temperance society, because it is an implied acknowledgment of the inefficiency of the Christian system to meet the emergencies of the age; whereas, I believe HIS "command is exceeding broad," embraces every good principle, and will reach every emergency of man, to the last hour of recorded time.

For outsiders, it may be proper enough to join a temperance society, founded on true principles; but for a church member to do so, is a degradation of his profession; for when a man professes to be a Christian, he professes to be and to do all that he is capable of, in the way of right, and higher than this he cannot go.

I do not think that it required much sagacity to foresee that drunkenness was not to be rooted out of the land by all the paraphernalia of badges, and pledges, and processions of tee-totallers, Washingtonians, "Sons," "Daughters," and the like. If we needed proof of all this, beyond what our own observation affords, the reported assertion of Gough, the Napoleon of temperance, is confirmatory, that of the five hundred thousand who have taken the pledge in the United States, four hundred and fifty thousand have violated it. Another great fact, elicited by the excise statistics of Ireland, is, that in eighteen hundred and fifty-three, more liquor was consumed in Ireland with a population of a little over six millions, than there was before Father Matthew administered the pledge to fanatical thousands, in a population of eight millions.

I lay it down as a universal and infallible fact, that *success in accomplishing any great reform is to be attained in one of two ways*—THE INDIVIDUAL MUST REFORM EITHER FROM A HIGH AND ABIDING SENSE OF RIGHT, OR FROM NECESSITY. Therefore, as to the temperance *purpose*, the end must be attained by the high sense of right, on the part of Christians and others of high moral principles, and by the "*necessity*" on the part of all others, and that necessity is fundamentally

THE MAINE LAW.

I believe that, at this age of the world, nothing can eradicate drunkenness and its myriads of woes but the Maine Law, in essence; and I believe its enactment and execution will. When the millennium comes, that is, when true religion generally prevails, no Maine Law will be needed.

While writing the above, the inquiry came across me, will not these announcements lose me subscribers? Styling it "anti-Christian for a member of the church to join a temperance society?" A classmate of mine, who sits in his little sanctum, and wields an influence throughout this wide republic, and will leave it to be felt long after he has passed away, said in a confiding hour, "I am afraid to publish all that I believe to be true." I wondered at it, too, for I knew him, just out of his teens, to brave the indignation of a class of three hundred young men, rather than make a pledge to some principle of a secret organization which he did not consider right. Has battling with the cold, hard world bowed his proud young heart; or has age brought with it its "*fears in the way*," its "*policy*," and its concealments? Alas, that it should be so! that we cannot remain through age, and penury, and sickness, and to the latest hour of life, the same proud, fearless, independent spirits we were, when glorious youth was ours.

If fearing to say what we believe to be true, in a proper place, time and manner, is a sign of age, then am I not old, then on the contrary am I growing younger, for constantly some new shackle is falling off me, and constantly is public opinion having less influence over me, because I have known public opinion to be wrong, and it was one of the secret promptings to publish this journal, to call in question some of these public opinions, for the purpose, not of denouncing, but of stimulating scientific reinvestigation as to health, bodily, mental, social.

Before this temperance digression we were legitimately discussing the subject of drinks in relation to health, and were saying that men had been led astray as to use of tea and coffee as they had been led astray as to other things, giving temperance as an example, that it had ran off into fanaticism in consequence of the ignorance of some of its over zealous unfledged advocates. The grand starting point of all the friends of "Temperance" so called, is that "a mere stimulus is useless and pernicious, coffee is nothing more than a mere stimulus, tea is nothing more than a mere stimulus: therefore, tea and coffee are useless and pernicious; it is a sin to practice what is pernicious, therefore the use of tea and coffee is a sin: no christian can possibly live in the habitual commission of any sin, therefore, to use tea and coffee is unchristian," so runs the argument as to the use of ardent spirits, and so as to slavery. However conclusive this mode of reasoning may be as to rum and slavery, it is evidently the reverse as to the use of tea and coffee, and demonstrably so, because the premises are false as to fact; hence all that follows, fails.

"Once upon a time," not very long ago, a party of men left Salt Lake City for St. Louis, with the United States mail, to be delivered at Independence or "*St. Joe.*" It was winter. They found the prairies covered with snow, and finally their "*animals*" perished with hunger; at this stage the six men found themselves utterly destitute of food; the game had taken to the woods, there were no rivers, the ground was covered with snow, they were still hundreds of miles from their journey's end, while the bleak winter winds whistling across the wide prairies in unobstructed fury, froze them sometimes almost to the heart's core. All, absolutely all they had to subsist upon under these desperate circumstances, was snow water and a quantity of green coffee; this they burned and boiled in snow water, and upon it travelled for six days, until they reached a place of help. These are the bare facts of the case, as reported to government, and demonstrate that coffee, alone, is a sustenant, as well as a stimulant, that it contains the elements of nutrition, consequently it is not a mere stimulant, and all that has been said of "mere stimulants," is not applicable to it. Coffee then being of itself nutritious, capable of sustaining life for days at a time, under circumstances of severe cold and the labor of travelling on foot, and it being customary to use it with cream and sugar, which are themselves concentrated nutrients, and withal, being drank hot, the conclusion appears to us legitimate as one of Euclid's corollaries, that *coffee as generally used in this country is a valuable, nutritious, healthful and comfortable item.*

Chemical analysis, has of late, under the direction of the most competent and intelligent minds of the age, arrived at the point just stated, and declares that coffee is a nutrient and that its essential principle, although one hundred and twenty-five per cent. less, is identical with that of the tea of commerce; and when facts, universal custom, and science, all unite in one point, surely we may feel safe, and hereafter take our cup of coffee and tea "in peace and quietness."

Reader, will you not show your gratitude for this comforting announcement by sending, by return mail, one dollar for the Journal for eighteen hundred and fifty-five, and another dollar for it to be sent to the address of your clergyman? We have other as comfortable announcements to make to you in the progress of the next year, but the January number of the Journal must have a messenger dollar or you will never see it more, out of New York city.

Having said so much about a cup of tea and coffee, it is proper to say something of its preparation. Individuals and nations have their preferences, but some things must be laid down as of universal application:

The first cup of coffee is the best.

The last cup of tea is the best.

Never take more than one cup at a meal.

Never increase the strength.

If it were a *mere* stimulant, then, after a while, it might, if not increased in strength or quantity, produce no sensible effect, might do no good, as brandy, opium or any other mere stimulant; but as tea and coffee are nutritious, the more so as they are used with milk and sugar, a cup of the "*self same*" is likely to do you as much good and as little harm twenty years hence as to-day.

It has been justly said that "In the life of most persons a period arrives when the stomach no longer digests enough of the ordinary elements of food to make up for the natural daily waste of the bodily substance. The size and weight of the body, therefore, begin to diminish more or less perceptibly. At this period tea comes in as a medicine to arrest the waste, to keep the body from falling away so fast, and thus enable the less energetic powers of digestion still to supply as much as is needed to repair the wear and tear of the solid tissues. No wonder, therefore, that tea should be a favorite, on the one hand, with the poor, whose supply of substantial food is scanty, and on the other way the aged and infirm, especially of the feebler sex, whose powers of digestion and whose bodily substance have together begun to fail. Nor is it surprising that the aged female, who has barely enough of weekly income to buy what are called the common necessaries of life, should yet spend a portion of her small gains in purchasing her ounce of tea. She can live quite as well on less common food when she takes her tea along with it; while she feels lighter, at the same time more cheerful and fitter for her work, because of the indulgence.

The use of tea became general in China about the year six hundred, A. D., and after a dozen hundred years' use, they seem to live as long as the Anglo-Saxons do, with whom, a thousand years later, it was so costly, that the East India Company considered the present of two pounds of it to the Queen of England a rare gift; and now, the average length of life in Great Britain is greater than when that present was made, although the inhabitants consume fifty-five million pounds of tea every year.

The effect of tea is to enliven; it produces a comfortable exhilaration of spirits, it wakens up, and increases the working capabilities of the brain, and brings out the kindlier feelings of our nature in moderation, having them always under our control. Alcohol, in any of its combinations, intoxicates, makes wild, places a man out of his own power, he gets beside himself, he can't control himself, nor can any one else control him, except by brute force. Upon some persons it has the effect of eliciting the darkest and deadliest passions of our nature. Whoever heard of a cup of tea inciting its sippers to "treasons, stratagems and spoils?" In certain irritated states of the body, it soothes the whole system, allays inflammation, cools fever, modifies the circulation, and counteracts the stupor of opium and brandy.

### HOW THE CHINESE USE TEA.

They put a few leaves in a porcelain cup, pour boiling water upon it, and cover the cup with a lid; in about a minute depress one edge of the lid, to keep the leaves in, and sip the tea: the moment the cup is emptied, the cup is filled with hot water. This refilling is continued at pleasure, without putting in more leaves. One may in this manner have several nice cups of tea, from one supply of the leaf. The Chinese add nothing to their tea. The English added cream and sugar, at first, to make it more palatable; this, in time, became a custom—the addition, however, by imparting increased nourishment, adds to its value as an item of daily diet.

The crusader against tea and coffee will tell you that the main element of both is composed of the same constituents as strychnine, a sixth of a grain of which will kill a dog in half a minute, while less than a grain will kill a man. The main elements of strychnine and the elements of the main principle of tea and coffee are oxygen, nitrogen, hydrogen and carbon, but these elements are in different proportions, and that makes all the difference in the world; for, as was intimated before, the air we breathe is composed of nitrogen and oxygen, and so is aqua fortis, but in different proportions, with water added. Alcohol is composed of oxygen, carbon and hydrogen, and so is sugar. Morphine, a dozen grains of which will kill a man in a few hours, is composed of nitrogen, carbon and hydrogen, and so is the extract of tea; but the different proportions of the ingredients change the very nature of the product; hence we are not to judge of the wholesomeness of an article from its elementary constituents, but from its observed effects on the system in the course of a life time, or a generation; and as the average duration of human life has been considerably lengthened, notwithstanding the enormous increased consumption of tea and coffee during the same time, their general effect on the human system is not certainly discouraging.

No doubt some persons are injured by the use of tea and coffee, but to argue that because one in a million is injured, the remainder of the million must also be injured, and should therefore forego its agreeable effects, is a tyranny not to be submitted to; it is a positive folly, especially when it is quite certain, that the very persons who are injured by it, are those who have abused its use; and to reject an article of food or drink because its use may be abused, and such abuse lead to disastrous results, is simply ridiculous.

### TEA AS FOOD.

Through all the wastes of Asia, the use of tea is universal; not its infusion, as with us, but the leaves are matted together like flaxseed oil cake, and as hard almost as a piece of wood; these hard cakes or balls, when wanted, are dissolved in water, then mixed with the blood of animals, enriched with the fat of beef or mutton, and then eaten with a spoon like thick soup.

### THE TIME TO DRINK TEA

is at supper, when the lightest meal of the day is taken; for, by its exhilarating effects, it destroys the sense of hunger, and enables a person to go to sleep without having much in the stomach to keep it working all night, and so prevent sound refreshing sleep.

One of the great secrets of health is a light supper, and yet it is a great self-denial, when one is hungry and tired at the close of the day, to eat little or nothing; let such an one take leisurely a single cup of tea and a piece of cold bread with butter, and he will leave the table as fully pleased with himself and all the world, as if he had eaten a heavy meal, and be tenfold the better for it next morning. Take any two men under similar circumstances, strong, hard working men, of twenty-five years; let one take his bread and butter with a cup of tea, and the other a hearty meal of meat, bread, potatoes, and the ordinary et ceteras, as the last meal of the day, and I will venture to affirm, that the tea-drinker will outlive the other by thirty years.

### TO MAKE A CUP OF TEA.

The teapot itself should be as perfectly plain and even in shape, inside and out, as possible; it will thus throw off less heat, and consequently keep hot longer, and be more easily kept thoroughly clean. A level teaspoon for one cup.

When the pot is perfectly clean, and dry, put the dry tea in and stand it before the fire for at least ten minutes; then pour on the boiling rain or soft water, let it stand five minutes, and it is ready for use; then put your sugar and milk in the tea-cup, and pour the tea upon it.

### TO MAKE A CUP OF COFFEE.

As soon as the coffee is parched, scarcely brown, grind as much as you will want to use for that time; put it in the coffee-pot, and pour on boiling water; stir, place it on the fire, bring it to a boil; as soon as four or five bubbles appear, take it off the fire, pour out a tea-cupful and return it, then set the pot down for one minute, next pour gently over it a tea-cup of cold water, let it stand another minute to allow the heavy cold water to sink to the bottom and carry the grounds with it, then put your usual amount of sugar in your coffee-cup, and as much boiling milk as you desire, then fill it from the coffee-pot.

### A FRENCH CUP OF COFFEE.

When in Paris in eighteen hundred and forty-four, I learned the French method of preparing coffee, and prefer it to any other.

Imagine a large tin pepper-box inverted with the bottom knocked out: put into this as much coffee as you desire, hold it over your coffee-pot, which should be large enough in circumference to receive about an inch of the quondam pepper-box, pour on boiling water in a stream as large as a common quill, and as soon as the water has passed through the ground coffee, it is fit for use without any special need of clearing.

As much ground coffee as can be taken up with a dessert spoon is sufficient for one person.

As coffee, when roasted, ground and exposed in an open vessel, is a more powerful deodorizer than chloride of lime, without its disagreeable smell, it is reasonable to conclude that it will act in the same manner in the human stomach, and by antagonizing disagreeable odors there, would remove foul breath.

### A SUBSTITUTE FOR COFFEE.

From chemical analysis it appears that the seeds of the Asparagus when dried, parched and ground, make a full flavored coffee, but little inferior to Mocha, containing in common with tea and coffee, the principle called taurine. Dry the asparagus berries well, after being thoroughly ripened, then rub them on a sieve, thus the seeds are readily separated.

While on this subject of aliment I wish to make some suggestions as to the quality of certain articles of food, their preservation, cooking, &c., for there is perhaps even more in preparing food for the table, than in the quality itself.

### HOW TO COOK A POTATO.

Wipe it carefully without breaking the skin, and cut from the thickest end a piece the size of a dime, and when the water is boiling, put in the potatoes. If the potatoes are watery put a piece of fresh lime in the water, as large as an English walnut to each dozen of good sized potatoes, then bring the water to a boil, put in the potatoes with the skin on as before, and in either case they will come out perfectly dry and mealy, if as soon as they are sufficiently boiled they are removed from the water and placed on the dish for the table, without covering them for a single second.

### BROILED POTATOES.

Cut cold boiled potatoes in slices a quarter of an inch thick, dip each slice in wheat flour, place them on a griddle over a fire of bright coals; when both sides are browned place the slices on a hot dish, and butter, pepper and salt to suit your taste, and place them hot on the table.

### FRIED POTATOES.

Wash, peel and slice quite thin, put, say a quart of these slices in a quart of lard as hot as it can be made without burning.

### TO PRESERVE CRANBERRIES.

As the cranberry is a delightful dish in winter, it may be well to know that if dried a short time in the sun and placed in bottles, filled with them, and then closed with sealing-wax, the berries will keep in good condition for several years.

### TO BAKE HAMS.

The usual mode of preparing hams for the table is by boiling; they are far richer if baked as follows:

Soak the ham in clean water for an hour, then wipe it dry, and spread it all over with thin batter, lay it in a deep dish with sticks under it to keep it from the grease. When fully done, remove the skin, and the batter which has crusted on the flesh side, and set it away to cool in the open air.

### PICKLES.

As it is now the pickling season, and pickles are a very healthful article of diet, it may be well to know how to prepare them properly. Some persons have a great horror of pickles, not that they have any reason for it beyond having a "*notion*" to that effect; this has possibly arisen from the fact that young girls not in good health relish them very much, and eat them in large quantities perhaps; the superficial observer then sets it down as a clearly established fact that the pickles are the cause of the ill health, while the fact is, that animal instinct called for something sour as a means of counteracting forming dyspepsia, calling for something sour to aid the stomach in performing its work of digestion, vinegar being more like the gastric juice in its properties than any other known fluid, hence delicate persons may take them to advantage at the close of a meal, not swallowing the solid parts.

Pickles should never be kept a moment in any vessel except it be of stone, wood, porcelain or glass; in most other vessels, earthen or metallic, they soon become poisonous.

To each three gallons of vinegar of moderate sharpness add a tea-spoonful of powdered alum and a tea-cup of common salt, then put into it a bag of pepper and such other spices as you desire, then it is ready to receive any kind of pickling; stir them occasionally, and if there are soft ones, take them out, scald the vinegar and pour it hot over the pickles, and always let the vinegar cover the pickles well. Vinegar should not be boiled longer than five minutes.

There are few subjects relating to health of greater importance than the proper regulation of the bowels without the use of medicine; the judicious use of fruits will accomplish this to a considerable extent in many persons; but there are times and places and seasons when ripe fruits, and fresh, are not attainable; and as those preserved in spirits or sweets do not answer the purpose, I here introduce an anonymous article, which embraces much useful information on the subject of

### PRESERVING FRUITS WITHOUT SUGAR.

We have received numerous applications for information about the *modus operandi* of putting up fruit so as to preserve it in a fresh state, without cooking, drying, or packing in sugar. It is a business that cannot so well be done in families as in large manufactories, where everything is arranged for convenience; but still with a little experience and careful attention, every family can save enough of the various fruits of the season to furnish their tables with a great delicacy during that portion of the year when they can get nothing of the kind. The whole secret consists in expelling the air from bottles or cans, by heat, and then sealing up the contents hermetically. If the article to be preserved is peaches, select such as you would for sweetmeats, and pare and cut them so they can be put in the bottle, and you must do this with the least possible delay, or they will be colored by the atmosphere. Some persons who want them to retain their natural whiteness, put them under water. When the bottle is full, cork it tight and wire down the cork with very little projection above the glass. When you have bottles enough to fill a kettle, such as may be most convenient, put them in and boil with the water all around up to the nozzle, for about fifteen or twenty minutes, or until the bottle appears to be full of steam—the atmosphere having been forced out through the cork. As soon as the bottles are cool enough to handle, dip the corks in sealing-wax so as to cover them quite tight. An additional precaution is used by some in putting tin foil over the wax.

Another plan is to cook the fruit slightly in a kettle, and then put it in cans or bottles, and pour hot syrup of sugar in to fill up the interstices, and then cork and seal. The heat of the fruit and syrup answering to expel the air. But the less they are cooked, or sweetened, the more natural will be the taste, like fresh fruit, when opened. We have eaten peaches a year old that we could not tell from those sugared an hour before.

Tomatoes are very easily preserved, and retain their freshness better than almost any other fruit. The small kind are only used. Scald and peel them without breaking the flesh. Bottles should hold about a quart only, because when once opened, the contents must be used up at once. Bottles made on purpose, with large throats, and a ring on the inside are the best, and are better than cans for all acid fruit. The cans, however, are more easily secured by solder than the bottles by corks and wax, as the air is let out through a small puncture after the large opening is soldered up and cans heated, and that hole stopped with a single drop of solder.

Every article of fruit will keep fresh if the air is exhausted and the bottle sealed *tight.* The least particle of air admitted through any imperfection of the sealing will spoil the fruit. If the air could be driven out without heat, there would be no need of any cooking, and only just enough should be given to expel the air and not change the taste. Many persons prefer to add syrup made by about one pound of sugar to a quart of water to all suitable fruits. Green corn, beans, peas, tomatoes, pie plant, currants, gooseberries, cherries, plums, raspberries, strawberries, peaches, are the most common things put up in this way. They add greatly to the pleasures of the table, and to the health of those who consume them; quite unlike, in that respect, the common preserves.

We have known fruit for pies put up in three-quart cans, by partially cooking in an open kettle, in a syrup just sweet enough for use, and putting the fruit in the cans hot and soldering immediately. It kept thus perfectly.

Some fruits keep much better and with less heating than others. Peas are among the hardest articles to keep; they contain so much fixed air.

We advise every family in the country to try this plan of putting up fruits for winter use, on a small scale this year, and if successful, enlarge upon it next year.

As an article of diet, as to its nutritive and medicinal qualities, not second to any known, is

### THE TOMATO.

To many persons there is something unpleasant, not to say disgusting, in the flavor of this excellent fruit. It has, however, long been used for culinary purposes in various countries of Europe, and has of late years been extensively cultivated, and become a general favorite in this country. Dr. Bennett, a professor of some celebrity, considers it an invaluable arti cle of diet, and ascribes to it very important medical properties. He declares:

1. That the tomato is one of the most powerful deobstruents of the *Materia Medica*, and that in all of those affections of the liver and other organs where calomel is indicated, it is probably the most effective and least harmful remedial agent known in the profession.

2. That a chemical extract will be obtained from it, which will altogether supersede the use of calomel in the cure of disease.

3. That he has successfully treated serious diarrhœa with this article alone.

4. That when used as an article of diet, it is almost a sovereign remedy for dyspepsia or indigestion.

5. That persons removing from the east or north to the south or west, should by all means make use of it as an aliment, as it would in that event, save them from the danger attendant upon those violent bilious attacks to which almost all unacclimated persons are liable.

6. That the citizens in ordinary should make use of it, either raw, cooked, or in the form of a catsup, with their daily food, as it is the most healthy article in the *Materia Alimentaria.*

Professor Rafinesque, of France, says, "It is every where deemed a very healthy vegetable, and an invaluable article of food."

Dungleson says, "It may be looked upon as one of the most wholesome and valuable esculents that belong to the vegetable kingdom."

Professor Dickens asserts that, "It may be considered more wholesome than any other acid sauce."

A writer in the Farmer's Register says, "It has been tried by several persons, with decided success. They were afflicted with chronic cough, the primary cause of which, in one case, was supposed to be diseased liver—in another, diseased lungs. It mitigates, and sometimes effectually checks, a fit of coughing."

The method most commonly adopted in preparing this fruit for daily use, is to cut them in slices, and serve with salt, pepper, and vinegar, as you do cucumbers.

To stew them, remove them ripe from the vines, slice up, and put them in a pot over the stove or fire, without water. Stew them slowly, and, when done, put in a small piece of good butter, and eat them as you do apple-sauce. Some add a little flour bread, finely crumbled, or a couple of crackers pulverized.

The tomato is a fruit very easily raised. If the seed be sown in May, in good rich soil, of a warm nature, with a sufficiency of old, well-rotted manure, there will rarely be any danger of failure. When the vines begin to lean, they should be provided with a trellis, or tied to stakes fixed in the soil, to keep the fruit from being injured by coming in contact with the dirt.

### PRESERVED NATURAL TOMATOES.

Pack them in jars—laying alternately a layer of tomatoes and a layer of sand, until the vessel is full. Cover them closely to prevent the introduction of air, and place them where they will remain cool. Thus packed, they will last a whole year.

### TOMATO KETCHUP.

The following, from long experience, we know to be the best receipt extant for making tomato ketchup:

Take one bushel of tomatoes, and boil them until they are soft. Squeeze them through a fine wire sieve, and add—

Half gallon of vinegar;
One pint and a half of salt;
Two ounces of cloves;
Quarter pound of allspice;
Three ounces of cayenne pepper;
Three table-spoonsful of black pepper;
Five heads of garlic, skinned and separated.

Mix together and boil about three hours, or until reduced to about one-half. Then bottle without straining.

### TOMATO FIGS.

We have seen several recipes for putting up tomato figs, but the following from the *Royal Gazette*—Bermuda—appears to rest upon very reliable authority, and is no doubt valuable. Let our lady readers preserve it until the coming tomato season, and then give it a trial.

It will be noticed that Mr. J. B. Heyl, chemist and druggist of this town, obtained a prize at the Show held at Mount Langton last Wednesday, for tomato figs. As the tomatos prepared according to Mr. Heyl's plan are very delicious, and may be made an article of export, we have obtained from him a copy of his recipe, which we subjoin for general information.

Take six pounds of sugar to one peck—or sixteen pounds—of the fruit, scald and remove the skin of the fruit in the usual way, cook them over a fire, their own juice being sufficient without the addition of water, until the sugar penetrates and they are clarified, they are then shaken out, spread on dishes, flattened, and dried in the sun. A small quantity of the syrup should be occasionally sprinkled over them whilst drying, after which pack them down in boxes, treating each layer with powdered sugar. The syrup is afterwards concentrated and bottled for use. They keep well from year to year, and retain their flavor surprisingly, which is nearly that of the best quality of fresh figs. The pear-shaped or single tomatos answer the purpose best. Ordinary brown sugar may be used, a large portion of which is retained in the syrup.

Between the opening of spring and the close of summer, looseness of the bowels is very prevalent, the employment of salted ham broiled, two or three times a week, in warm weather, is a preventive to a considerable extent, in persons of temperate regular habits; it becomes then a matter of interest to know of the best methods of

### CURING HAMS.

After the meat is cold, rub the flesh side well, and fill the hock with fine salt. Leave it in this state from one to three days, according to the size of the hams. Then pack in a cask, and make a pickle of salt and water, strong enough to bear a potato; of sufficient quantity to cover the meat. Add to this pickle four ounces of dissolved saltpetre, and two quarts of West India molasses, for every 100 pounds of meat, and then pour the liquid over the meat. Should you find the quantity of pickle too small, add enough strong salt and water to cover the meat.

Small pieces may be taken out at the end of a month, and smoked. Large pieces should be left in pickle six weeks.

When the hams are very large, a portion of the thigh bone should be removed before salting. The finest hams a refrom hogs not over one year old. Many persons are unable to dispose of the spareribs, feet, &c., when the weather is moderate. I put mine on the top of the hams, and pour the pickle over the whole. Then, towards spring, soak them a few hours in cold water before using, and we find them a delicacy rather than a drug.

There is a class of invalids and weakly persons who find a fresh egg, taken in the morning, both grateful and strengthening; to such, as well as families in general, it will be well to know

HOW TO KEEP EGGS FRESH.

As detailed in the *Maine Farmer*, the following very simple plan we have never tried, and know nothing practically whether it be effectual or not. We found it in the "Farm Journal," quoted from the "English Agricultural Gazette." We pass it over to our readers for their consideration.

Take a half inch board of any convenient length and breadth, and pierce it as full of holes, (each one and a half inches in diameter,) as you can. I find that a board two feet and six inches in length, and one foot wide, has five dozen in it, say twelve rows of five each.

Then take four strips two inches broad, and nail them together into a rectangular frame. Nail this board upon the frame, and the work is done, unless you choose to nail a beading around the top.

Put your eggs in this board as they come from the poultry house, the small end down, and they will keep good for six months, if you take the following precautions:—Take care that the eggs do not get wet, either in the nest or afterwards. (In summer, hens are fond of laying among the weeds or grass, and any eggs taken from such nests in wet weather, should be put away for immediate use.) Keep them in a cool room in summer, and out of the reach of frost in winter. If two boards be kept, one can be filling while the other is emptying.

The writer accounts for the preservation of eggs in this way, by supposing that the yolk floats more equally in the white, and has less tendency to sink down against the shell, than when the egg is laid on one side—certainly, if the yolk touches the shell it spoils immediately.

HOW TO COOK AN EGG.

An egg should not be boiled; it should only be scalded—*vulgo*, coddled. Immerse your egg in, or, what is better, pour upon your egg boiling water. For time, proportion the same to the size and number of your eggs, and the collateral accidents. If you cook your eggs upon the breakfast table, more time will be required. But if you station your apparatus on a good wholesome hob, where there is a fire, and so the radiation of heat is less positive, less time will suffice. The latter way is mine, winter and summer, and the difference of the surrounding circumstances equalize, or nearly so, the time. I keep one egg under water nine minutes; two, nine and a half; three, ten; and four, nearly eleven minutes. The yolk first owns the power of the caloric, and will be even firmly set, while the white will be milky, or at most tremulously gelatinous. The flavor superior to anything which a plover ever deposited will be that which the egg of the gallinaceous domestic was intended to have; the substance, that which is delectable to the palate, and easy of digestion. There is perfect absence of that gutta percha quality, in the white especially, at once the result and the source of dyspepsia. I believe that eggs would be much more patronized and much more wholesome, if boiling were discarded.

HOW TO PREPARE DIGESTIBLE TOAST.

A highly philosophical description is given in the *Household Almanac* for 1853, of the proper mode of toasting bread. It is as follows:—"Chestnut brown will be far too deep a color for good toast; the nearer you can keep it to a straw-color the more wholesome it will be. If you would have a slice of bread so toasted as to be pleasant to the palate, wholesome to the stomach, never let one particle of the surface be charred. To effect this is very obvious. It consists in keeping the bread at the proper distance from the fire, and exposing it to a proper heat for a due length of time. By this means the whole of the water may be evaporated out of it, and it may be changed from dough—which has always a tendency to undergo acetous fermentation, whether in the stomach or out of it—to the pure farina wheat, which is in itself one of the most wholesome species of food, not only for the strong and healthy, but for the delicate and diseased. As it is turned to farina, it is disintegrated, the tough and gluey nature is gone, every part can be penetrated; it is equally warm all over, and not so hot as to turn the butter into oil, which, even in the case of the best butter, is invariably turning a wholesome substance into a poison. The properly toasted slice of bread absorbs the butter, but does not convert it into oil; and both butter and farina are in a state of very minute division, the one serving to expose the other to the free action of the gastric fluid in the stomach; so that when a slice of toast is rightly prepared there is not a lighter article in the whole vocabulary of cookery."

Having said so much on the subject of edibles we will pass to other items, mentioning only the best method of

### PRESERVING GREEN CORN.

Gather the ears when in full milk, and strip off all but a thin covering of husk; lay these in a moderately heated oven or cooking-stove long enough to scald or stiffen the milk, when the grains are shaved off and kept in a close bag or canister. When wanted for use, in the season, boil it with the inner husk on.

